# The basolateral vesicle sorting machinery and basolateral proteins are recruited to the site of enteropathogenic *E*. *coli* microcolony growth at the apical membrane

**DOI:** 10.1371/journal.pone.0179122

**Published:** 2017-06-21

**Authors:** Gitte A. Pedersen, Helene H. Jensen, Anne-Sofie B. Schelde, Charlotte Toft, Hans N. Pedersen, Maj Ulrichsen, Frédéric H. Login, Manuel R. Amieva, Lene N. Nejsum

**Affiliations:** 1 Department of Clinical Medicine, Aarhus University, Aarhus, Denmark; 2 Department of Molecular Biology and Genetics, Aarhus University, Aarhus, Denmark; 3 Department of Microbiology and Immunology, Stanford University, Stanford, CA, United States of America; 4 Department of Pediatrics, Stanford University, Stanford, CA, United States of America; Universita degli Studi di Bari Aldo Moro, ITALY

## Abstract

Foodborne Enteropathogenic *Escherichia coli* (EPEC) infections of the small intestine cause diarrhea especially in children and are a major cause of childhood death in developing countries. EPEC infects the apical membrane of the epithelium of the small intestine by attaching, effacing the microvilli under the bacteria and then forming microcolonies on the cell surface. We first asked the question where on epithelial cells EPEC attaches and grows. Using models of polarized epithelial monolayers, we evaluated the sites of initial EPEC attachment to the apical membrane and found that EPEC preferentially attached over the cell-cell junctions and formed microcolonies preferentially where three cells come together at tricellular tight junctions. The ability of EPEC to adhere increased when host cell polarity was compromised yielding EPEC access to basolateral proteins. EPEC pedestals contain basolateral cytoskeletal proteins. Thus, we asked if attached EPEC causes reorganization the protein composition of the host cell plasma membrane at sites of microcolony formation. We found that EPEC microcolony growth at the apical membrane resulted in a local accumulation of basolateral plasma membrane proteins surrounding the microcolony. Basolateral marker protein aquaporin-3 localized to forming EPEC microcolonies. Components of the basolateral vesicle targeting machinery were re-routed. The Exocyst (Exo70) was recruited to individual EPEC as was the basolateral vesicle SNARE VAMP-3. Moreover, several Rab variants were also recruited to the infection site, and their dominant-negative equivalents were not. To quantitatively study the recruitment of basolateral proteins, we created a pulse of the temperature sensitive basolateral VSVG, VSVG3-SP-GFP, from the trans-Golgi Network. We found that after release from the TGN, significantly more VSVG3-SP-GFP accumulated at the site of microcolony growth than on equivalent membrane regions of uninfected cells. This suggests that trafficking of vesicles destined for the basolateral membrane are redirected to the apical site of microcolony growth. Thus, in addition to disrupting host cell fence function, local host cell plasma membrane protein composition is changed by altered protein trafficking and recruitment of basolateral proteins to the apical microcolony. This may aid EPEC attachment and subsequent microcolony growth.

## Introduction

Enteropathogenic *E*. *coli* (EPEC) is a food-borne pathogen which can cause diarrhea and is annually responsible for thousands of deaths among infants in developing countries [1]. EPEC is also highly related to the most common strains of STEC (Shiga-toxigenic *E*. *coli*) which infect the host with similar mechanisms but also deliver a systemic toxin that can lead to life-threatening diseases including haemolytic uraemic syndrome [2, 3]. EPEC and STEC are not invasive bacteria, but rather attach to and replicate on the surface of epithelial cells of the small intestine. A major research focus is on dissecting the molecular mechanisms of EPEC and STEC interactions with epithelial cells to potentially prevent epithelial colonization and reduce morbidity caused by these organisms. From the bacterial perspective, an important unanswered question is whether EPEC alters the epithelial surface at the site of attachment to better colonize this niche.

EPEC triggers host cell changes including effacement of microvilli, mitochondria dysfunction [4, 5], inhibition of nutrient/water transporter function [6], modulation of inflammatory responses [7], inhibition of apoptosis [8], inhibition of phagocytosis [9, 10], and tight junction disruption [11, 12].

In epithelial cells, tight junctions separate the apical and basolateral plasma membrane domains. Thus, they ensure cell polarization and selective transepithelial vectoral transport of solutes and nutrients (for review see [13]). Many pathogens are known to disrupt tight junctions including *Helicobacter pylori* [14], *Vibrio cholera* [15], and *Clostridium difficile* [14–18]. Perturbation of cell junctions and cell polarity may enhance bacterial survival on the apical surface by facilitating the acquisition of nutrients, increasing the number of sites for adhesion, or subverting clearance mechanisms. It has been shown that disruption of cell polarity upon infection provides *H*. *pylori* access to iron and other nutrients from the basolateral side of the host cell, which was vital for the ability of *H*. *pylori* to grow and colonize epithelial cells [19].Whether EPEC perturbs cell polarity to access nutrition from the basolateral side is unknown.

Several mucosal bacterial pathogens can alter regulation of protein sorting leading to the recruitment of proteins associated with the basolateral membrane to the apical surface [20–22]. For instance, *Pseudomonas aeruginosa* has an affinity for basolateral proteins and recruits them to the apical attachment site [20, 21]. Also, *H*. *pylori* perturbs cell polarity by recruiting basolateral proteins to the site of bacterial attachment on the (apical) cell surface [22]. Thus, cell polarity remodeling may be a strategy used by bacterial pathogens during epithelial infection.

Successful infection by EPEC is dependent upon initial attachment followed by tight anchoring of EPEC to the apical surface of the host cell [23–25]. It has been suggested that EPEC may utilize host proteins as alternative receptors for attachment, including integrin β1 [26] which is a dedicated basolateral adhesion protein. Colonization of the cell surface is accompanied by translocation of several bacterial effectors into the host cell by the type 3 secretion system (T3SS). By directly altering intracellular signaling pathways, the effectors modify the membrane and cortical cytoskeleton at the site of adhesion. The first T3S-effector injected is the translocated intimin receptor (Tir), a transmembrane protein, which is inserted into the host cell plasma membrane. Once embedded in the plasma membrane, Tir functions as a high affinity receptor for intimin–a surface exposed adhesin that is anchored into the bacterial outer membrane [27]. Thus, the tight anchorage of EPEC is ensured by the delivery of its own receptor into the host cell. In the host cell cytoplasm, Tir is phosphorylated by host cell kinases, an event that initiates Tir signaling. This affects actin polymerization by recruiting specific adaptor proteins and actin regulators [28, 29]. The high rate of actin polymerization exerts pressure on the membrane beneath the EPEC attachment site and, consequently, the membrane protrudes forming a pedestal containing a core of actin underneath EPEC.

Several cytoskeleton-associated proteins are recruited to the actin pedestal, many of which are proteins normally associated with focal adhesions, including α-actinin, talin and vinculin [30–34]. In polarized colon adenocarcinoma T84 cells, EPEC infection for 6 hrs caused loss of tight junction, fence function and disruption of host cell polarity, where basolateral plasma membrane proteins were found in discrete puncta in the apical plasma membrane domain [35]. However, it is unclear if the appearance of basolateral proteins in the apical membrane is exclusively due to a loss of fence function, or if EPEC is also able to alter host cell vesicle trafficking and delivery upon infection. Moreover, it is unknown if the plasma membrane composition surrounding individual EPEC and EPEC microcolonies reflects apical or basolateral membrane composition, or a mixture.

In the present study, we found that access to basolateral membranes increased EPEC infection efficiency. We also observed that components of the basolateral vesicle sorting machinery localized to the site of infection at the apical membrane. Moreover, our data showed that basolateral membrane proteins concentrated around individual EPEC at the microcolony and that a basolateral protein, VSVG, localized around individual EPEC rapidly following release from the TGN. Thus, our results suggest that basolateral vesicle trafficking is redirected to the site of microcolony growth at the apical membrane. Here, the basolateral proteins localize around individual EPEC in the microcolony. Localization of basolateral proteins at the apical membrane upon infection may be due to a combination of a loss of fence function and a redirection of basolateral transport vesicles.

## Results

### Access to basolateral membrane proteins increases EPEC infection efficiency

EPEC infection is known to disrupt tight and adherens junctions [36, 37], which can compromize the fence function of the junctions and allow intermixing of apical and basolateral proteins in the membrane domains. Basolateral proteins have been identified at the apical membrane domain of infected cells [35], which could be caused by a loss of epithelial fence function. An alternative possibility is that the bacteria locally alter cell polarity at sites of attachment and microcolony growth. Thus, we investigated whether EPEC infection benefits from encountering basolateral membranes, and if local apical-basal host-cell polarity at the infection site is altered.

To study potential local effects of EPEC infection on the host-cell polarity, Madin-Darby canine kidney (MDCK) cells were used as a model system. MDCK cells are the most commonly used model for polarized epithelial cells in culture. Since they are not derived from a carcinoma, they do not have intrinsic defects in cell polarity, they form tight monolayers, and are easily polarized when grown on filter supports. Moreover, stable expression of fluorescently tagged proteins of interest can easily be generated in MDCK cells. Fluorescently tagged proteins can be used to visualize protein localization and, importantly for this study, to follow the timing of potential protein re-distribution during EPEC infection using time-lapse imaging.

**Fig 1:**
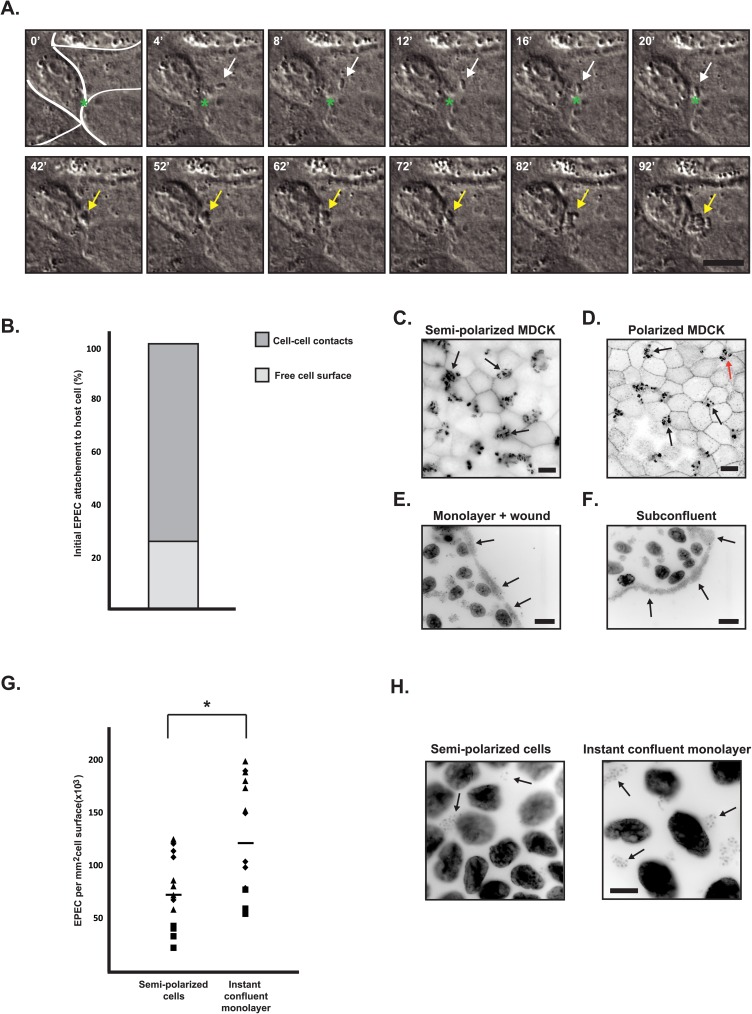
EPEC preferentially attached to cell-cell junctions and infection efficiency increased with access to basolateral proteins. **A-B**. Semipolarized monolayers of MDCK cells were infected with EPEC, and EPEC adherence to the cells was followed by live microscopy. A. Timelapse DIC imaging of attachment of an EPEC bacterium (indicated by white arrows) to the surface of a cell, movement to a tricellular cell junction (indicated by green asterisk), and establishment of a microcolony (indicated by yellow arrows). The full movie is shown in S1 Movie. The localization of initial EPEC adherence was evaluated from three or more different time-lapse sequences, and it was observed that 26 attachment events were on the free cell surface, and 76 attachment events were at cell-cell contacts (B). **C-D.** MDCK cells were seeded as monolayers for 3 days on collagen-coated glass (C) or semi-permeable filter supports (D) to allow the cells to polarize. The cells were infected with EPEC for 4 and 6 hours, respectively. They were then fixed and stained with phalloidin-rhodamine to visualize EPEC actin pedestals. Infections were established on top of tTJs (black arrows) and multicullular junctions (red arrow) (C and D). **E-F.** Cells grown 3 days on glass followed by the generation of a wound (E) or at subconfluence (F) were infected with EPEC for 4 hours.They were then fixed and stained with hoechst (E and F) to label both cell nuclei and EPEC bacteria. Increased numbers of EPEC were seen at the edges of the cell sheet where basolateral membranes were exposed (E and F). Scale bars are 10 μm (A, C and D) and 20 μm (E and F). **G.** Quantification of infecting EPEC on instant confluent monolayers versus semi-polarized monolayers. The cells were infected with EPEC for 4 hours and then fixed and stained with hoechst. The number of bacteria was counted from fluorescence microscopy images. Data points represent a total of 14–15 images from three data-sets; squares, diamonds and triangles represent counts from different data-sets. The difference was statistically significant with Student’s t-test. **H**: Representative examples of images used for quantification in G. Arrows indicate examples of bacteria. Scale bar is 10 μm. Fluorescence images are shown in inverted contrast.

To evaluate if EPEC attached evenly to the membranes of epithelial cells or if there was a preference towards a specific plasma membrane domain, infection of monolayers was performed directly at the time-lapse microscope. Fig 1A shows an example of an individual EPEC, which attached at or close to a cell-cell junction. Following attachment, the bacteria moved to a tricellular tight junction (tTJ, Fig 1A asterisk, and S1 Movie), where colony formation occured. Quantitatively, EPEC attachment occurred preferentially over the cell-cell junctions and not the free cell surface (Fig 1B). 102 individual attachment events from three different time-lapse experiments were quantified. Of these, 76 initial attachment events occurred on the cell-cell junctions, and 26 events were observed on the free cell surface. To test whether the preference for attachment to cell junctions is driven by a tropism towards basolateral proteins, infections at different stages of MDCK cell surface polarization were performed.

MDCK cells grown on glass coverslips for three days form a semi-polarized monolayer, in which the apical and basolateral membrane components are largely separated into domains by tight junctions, which are, however, slightly leaky (S1 Fig) [38–40]. When semi-polarized cells were infected, the majority of EPEC microcolonies were localized at tTJs (Fig 1C, black arrows). To create fully polarized monolayers, MDCK cells were grown on semi-permeable filter supports allowing formation of tight junctions and a complete separation of apical and basolateral proteins into their respective membrane domains [41, 42]. The transepithelial resistance (TER) was 210–235 Ω/cm^2^, consistent with previous reported TER measurements of polarized MDCK II cells [43]. After infection of polarized MDCK cells, the majority of EPEC microcolonies were localized at tTJs and multicellular junctions (Fig 1D, black arrows and red arrow, respectively), similarly to infection of the semi-polarized cells. Interestingly, EPEC infection of either a wounded cell monolayer or a subconfluent culture (Fig 1E and 1F, respectively) revealed that microcolonies were mainly formed at the edges of the cell layer adjacent to the substratum. Subconfluent MDCK cells or recently wounded monolayers yield access to basolateral proteins at the edges or at the wound edge, respectively (S1 Fig). Thus, these observations strongly suggest that EPEC preferentially forms microcolonies on membrane regions containing basolateral proteins.

To further confirm the tropism of EPEC for basolateral membrane proteins, we compared EPEC infection efficiency on two different MDCK monolayer types. Semi-polarized MDCK monolayers with partially separated apical and basolateral proteins were compared to instant confluent monolayers in which apical and basolateral proteins are mixed over the cell surface (S1 Fig) [40, 44, 45].

We quantified the infection efficiency from fluorescence microscopy images of instant confluent monolayers and three-day semi-polarized monolayers infected with equal amounts of EPEC. Since MDCK cells in three-day monolayers were more densely packed than in instant confluent monolayers (Fig 1H), we quantified the number of EPEC per area and not per cell. It was observed that 67% more bacteria established infection on instant confluent monolayers where basolateral proteins were readily accessible compared to three-day monolayers (Fig 1G), (p<0.05). Moreover, upon infection of fully polarized cells that were grown on filters for four days, EPEC formed small and confined colonies while large and irregular colonies were observed on instant confluent monolayers seeded on filter supports (S2 Fig).Together, these results support our hypothesis that EPEC infects MDCK cells with greater efficiently when basolateral membrane proteins are more accessible. Also, these data suggest that the presence of basolateral membrane proteins may enhance EPEC attachment to that region of the host cell membrane.

### Basolateral proteins are redirected to the apical membrane at the infection site

The data presented above strongly indicate that EPEC microcolony growth increased with access to basolateral membrane components. Therefore, we tested if local apical-basal host-cell polarity was altered at the site of microcolony growth which possibly could generate new and/or improved sites for further development of the infection.

To investigate whether basolateral proteins accumulate at the site of microcolony growth, the localization of basolateral proteins not known to be involved in establishment of epithelial polarity or EPEC attachment was investigated.

Aquaporin-3 (AQP3) is a water channel protein that has previously been used as a model protein to study trafficking and localization of basolateral membrane proteins [46, 47]. There are no reports of AQP3 involvement in EPEC attachment or pedestal formation.

**Fig 2:**
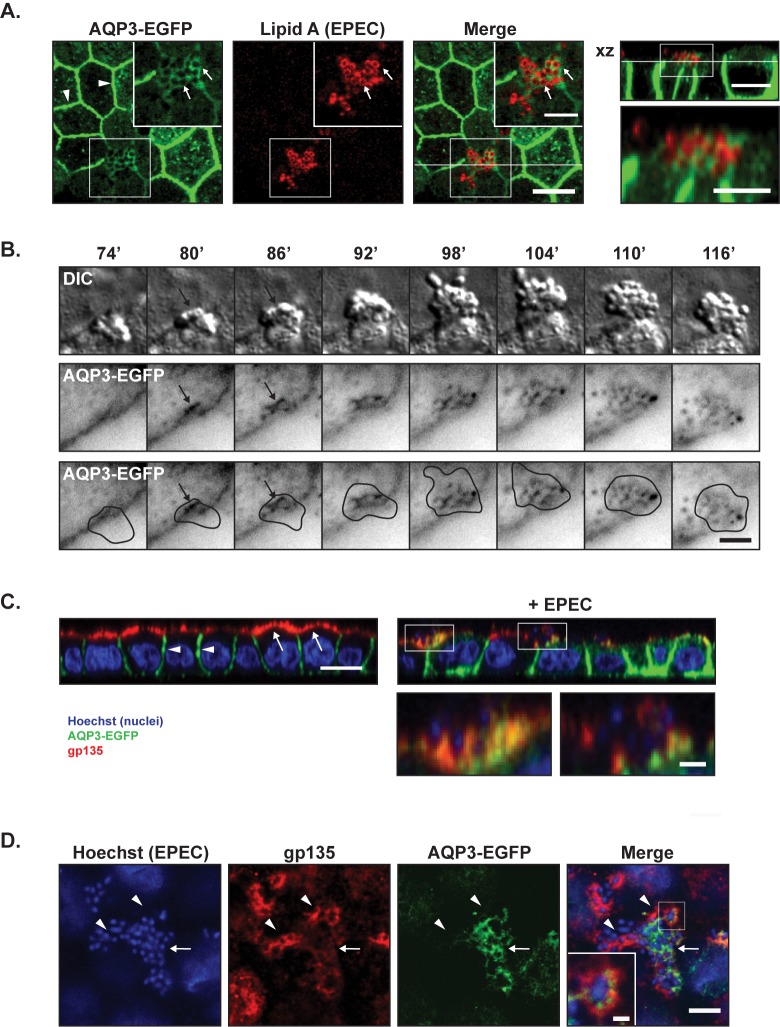
Basolateral AQP3-EGFP localized to the center of EPEC microcolonies, whereas apical gp135 localized to the periphery. **A.** MDCK-AQP3-EGFP cells were seeded to confluency on semi-permeable collagen-coated Transwell filter supports and allowed to polarize for 3 days. Cells were then infected with EPEC for 6 hours, fixed and stained with an antibody against Lipid A to label EPEC bacteria (shown in red). The rightmost image shows a xz view at the position indicated by the white line in the merge image. AQP3-EGFP localized to the lateral membrane (arrow heads) and also accumulated at the site of microcolony formation around individual EPEC bacteria (arrows). Scale bars: 10 μm and 5 μm (insets). **B.** MDCK-AQP3-EGFP cells were infected with EPEC bacteria directly into the heating chamber after mouting on the microscope. Time-lapse imaging was performed with 1 minute intervals for DIC (EPEC and cells) and EGFP (AQP3-EGFP). Montage shows DIC and AQP3-EGFP in inverted contrast. The bottom panel shows the EGFP image including a drawn outline of the bacterial microcolony based on the DIC image. AQP3-EGFP recruitment was observed at the center of microcolony formation (at approximately 80’ after initial attachment) and was sustained to the center of the microcolony with no detectable recruitment to the periphery of the EPEC colony. Scale bar: 5 μm. **C-D.** Polarized MDCK-AQP3-EGFP monolayers with and without EPEC infection for 6 hours were stained with a monoclonal anti-gp135 antibody (red) and hoechst (blue, to detect EPEC and cell nuclei). **C.** xz-projection of confocal z-stacks showed that AQP3-EGFP was localized to the basolateral membrane (white arrow heads), whereas gp135 was strictly localized to the apical membrane (white arrows) in the uninfected cells. Both AQP3-EGFP and gp135 were localized at the infection site in EPEC-infected cells. Scale bar: 10 μm and 3 μm for insets. **D.** xy maximum projection of 6 slices from a z-stack showing an EPEC microcolony. Arrows point to AQP3-EGFP, arrowheads to gp135. Gp135 seemed less intense at sites of AQP3-EGFP accumulation. Scale bar: 10 μm and 1 μm for the inset.

EPEC infection of polarized MDCK-AQP3-EGFP cells showed that AQP3-EGFP was recruited to the infection site on the apical surface (Fig 2A). Similarly, the transferrin receptor (TfR), which is predominantly localized to basolateral membranes and recycling endosomes, was also recruited to the EPEC infection site (S3 Fig).

To determine the kinetics of AQP3-EGFP recruitment to the infection site, time-lapse imaging of infected MDCK-AQP3-EGFP cells was performed. Time-lapse imaging revealed that AQP3-EGFP recruitment occured continuously following bacterial adhesion to the apical surface (Fig 2B).

Gp135/podocalyxin is a strictly apical protein and has been used previously for labeling the apical membrane of MDCK cells [48, 49]. Upon infection, gp135 localization was more heterogeneous, and gp135 was localized to infecting EPEC (Fig 2C). There was not complete co-localization of the recruited basolateral AQP3-EGFP and localization of the apical gp135 at the infection site (Fig 2D).

Taken together, these data indicate that basolateral proteins are recruited to the infection site on the apical surface as the microcolony grows. Thus our data strongly indicate a local remodeling of plasma membrane polarity as well as a loss of host cell fence function.

### Functional, but not dominant-negative, Rab proteins localized at the infection site

**Fig 3:**
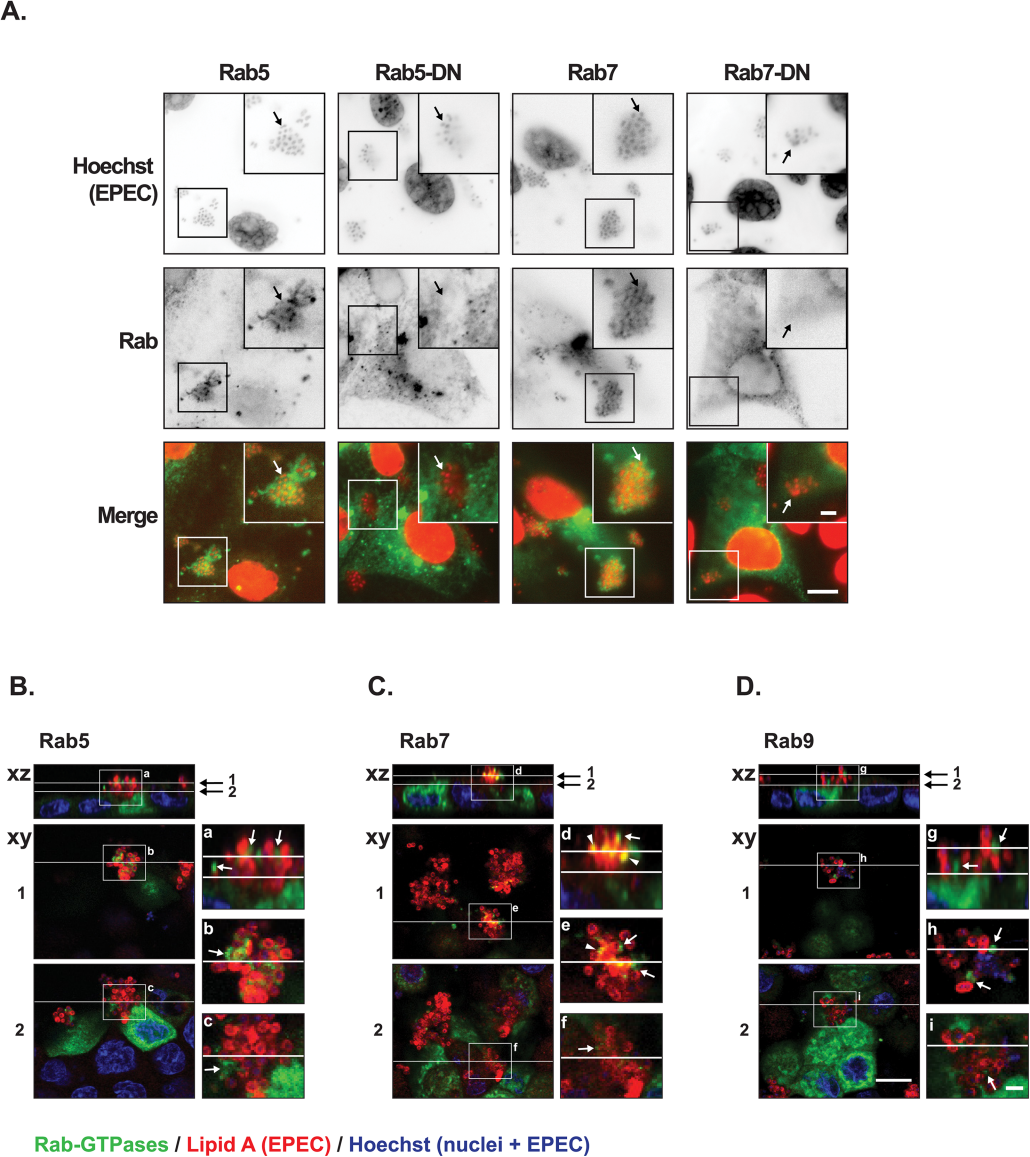
Endosome markers Rab5, -7 and -9, but not dominant-negative Rab5 and Rab7 were recruited to EPEC microcolonies. **A.** Subconfluent MDCK cells transiently transfected with Rab5-GFP, Rab5-DN-mCherry, Rab7-GFP, or Rab7-DN-mCherry were infected with EPEC for 4 hours, fixed and stained with hoechst to label cell nuclei and EPEC bacteria. All Rab proteins are shown as green in merge, and hoechst is shown in red. In upper panels, the individual channels are shown as inverted contrast. Rab5-GFP and Rab7-GFP localized at the EPEC infection site, whereas the dominant-negative variants Rab5-DN-mCherry and Rab7-DN-mCherry were deprived from the regions of EPEC infection. Arrows indicate examples of EPEC bacteria. Scale bar: 10 μm and 3 μm for inserts. **B-D.** Polarized MDCK cells transiently transfected with Rab5-GFP (B), Rab7-GFP (C) or Rab9-GFP (D) were infected with EPEC for 6 hours, fixed and stained with a polyclonal anti-Lipid A antibody (red) to label the EPEC bacteria and hoechst to label cell nuclei as well as EPEC bacteria (blue). Rab5-GFP, Rab7-GFP, and Rab9-GFP all localized at the infection site (white arrows). Rab7-GFP localize very close to EPEC as overlay could be seen (yellow, arrow heads). Positions of xy sections and xz projections are indicated by white lines. Insets show xz sections (a, d, g) and xy sections of the top of a microcolony (b, e and h) and at the bottom of a microcolony close to the plasma membrane (c, f and i). Scale bars: 10 μm and 2 μm for insets.

Since basolateral AQP3 accumulated apically at the site of microcolony growth, we investigated if EPEC could manipulate host cell vesicle trafficking. First we tested the subcellular localization of two Rab GTPases involved in vesicular trafficking [50]: Rab5, Rab7 as well as their dominant-negative counterparts. Rab5 and Rab7 are markers of early and late endosomes, respectively [51, 52]. Rab5-GFP, Rab5-DN-mCherry, Rab7-GFP, and Rab7-DN-mCherry were transfected into subconfluent MDCK cells. In non-infected cells, all Rab variants were distributed throughout the cell (S4 Fig). However, upon EPEC infection, Rab5-GFP and Rab7-GFP were recruited to and localized around individual EPEC, whereas the dominant-negative variants were not localized to the infection site (Fig 3A). This suggests that functional Rab proteins that may be active in vesicular trafficking are specifically recruited to the infection site.

We asked whether Rab proteins could also be recruited to EPEC in cells with a higher degree of polarization. MDCK cells were grown for two days on filter supports after transient transfection with Rab5-GFP, Rab7-GFP, as well as Rab9-GFP, which is a marker of both late endosomes and retrograde Golgi vesicles [53]. Upon infection with EPEC all of these Rab GTPases were found at the sites of EPEC microcolony formation (Fig 3B–3D) where they localized in puncta in the apical membrane surrounding EPEC. Together, this relocalization indicates that EPEC specifically affects host cell vesicle trafficking components during infection. No change in the expression level of endogenous Rab5 was detected upon EPEC infection (immunoblotting, not shown).

### The Exocyst and the basolateral v-SNARE, VAMP-3, localize to the infection site at the apical membrane

**Fig 4:**
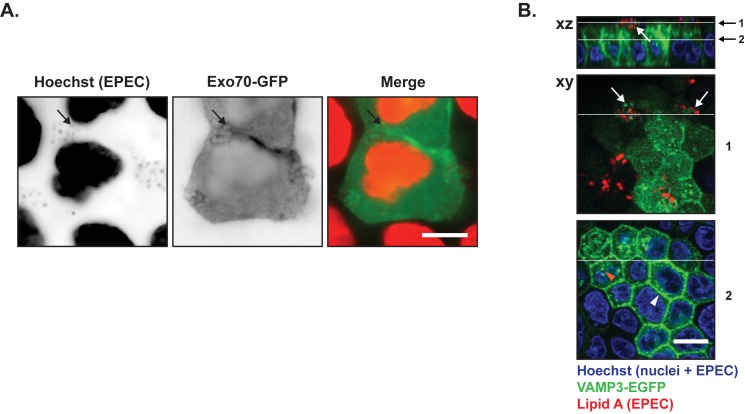
Components of the basolateral vesicle docking/fusion machinery were recruited to EPEC microcolonies. **A.** Subconfluent MDCK cells were transiently transfected with Exo70-GFP, which is part of the basolateral exocyst vesicle docking hub. The cells were infected with EPEC for 4 hours, fixed and stained with hoechst to label cell nuclei and EPEC bacteria. Hoechst labeling and Exo70-GFP are shown as inverted contrast and in red and green in merge, respectively. Arrows indicate examples of EPEC bacteria with Exo70-GFP localization. Scale bar: 10 μm **B.** Polarized MDCK-VAMP3-EGFP cells on semi-permeable Transwell filters were infected with EPEC for 6 hours, fixed and stained with anti-Lipid A to label EPEC bacteria (red) and hoechst to label cell nuclei as well as EPEC bacteria. In the basal region of the cell, VAMP3-EGFP localized at cell-cell contacts (white arrowhead) and as puncta in the cytosol (orange arrowhead). In the apical part of the cells,VAMP3-EGFP was concentrated at the sites of infection (white arrows). Positions of xy sections and xz projections are indicated by white lines, 1 is through an EPEC colony, 2 is close to the plasma membrane. Scale bar: 10 μm.

In polarized MDCK cells, the Exocyst localizes at the apex just beneath tight junctions [54, 55] where the complex tethers vesicles targeted to the basolateral membrane prior to their fusion *via* SNARE proteins [56]. Besides its role in exocytosis, the Exocyst has been suggested as a major regulator of endosomal trafficking for both apical and basolateral recycling and for basolateral-to-apical transcytosis [57]. To determine if the Exocyst complex was affected by EPEC infection, we transfected MDCK cells with Exo70-GFP; Exo70 is a major component of the Exocyst complex [47, 54, 55]. Exo70-GFP [58] localized primarily in cell-cell junctions (S5A Fig), however, after EPEC infection we observed that Exo70-GFP was recruited to the EPEC infection site where it localized around individual bacteria (Fig 4A). The basolateral v-SNARE protein VAMP-3 is involved in docking and fusion of vesicles containing basolateral cargo. Infection of cells stably expressing an EGFP-tagged version of VAMP-3 revealed that VAMP-3 also localized at the site of EPEC infection (Fig 4B, uninfected S5B Fig) in small, discrete puncta.

It is possible that the recruitment of Rab GTPases, the Exocyst complex as well as VAMP-3 to sites of EPEC microcolony growth corresponds to a rerouting of basolateral vesicles where the EPEC microcolonies may serve as a docking site for vesicles arriving from the basolateral surface or are redirected from the TGN.

### EPEC redirects basolateral proteins directly from the TGN

To test if vesicles with basolateral cargo are redirected to EPEC colonies directly from the trans-Golgi Network (TGN), we used a temperature sensitive version of VSVG, VSVG3-SP-GFP [59]. VSVG has a basolateral sorting signal [60] and is normally exclusively sorted to the basolateral membrane upon synthesis [61]. VSVG3-SP-GFP is misfolded and accumulates in the ER when cells are incubated at 40°C. By lowering the temperature to 20°C VSVG3-SP-GFP is realeased from the ER and accumulates in the TGN. Subsequently, VSVG3-SP-GFP can be released from the TGN at 37°C.

**Fig 5:**
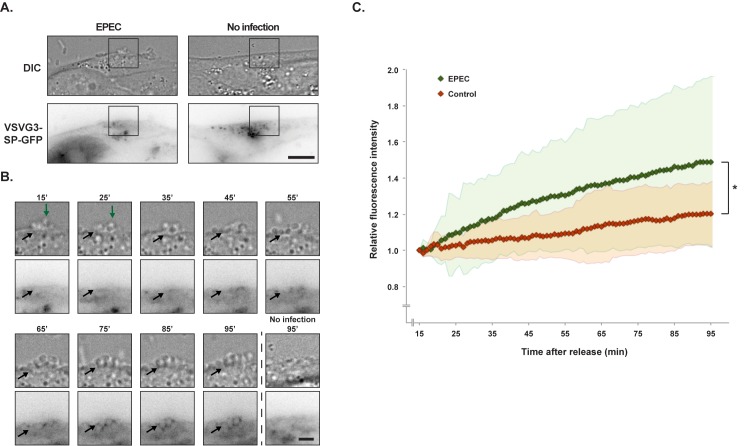
Freshly synthesized VSVG3-SP-GFP was recruited to the EPEC infection site. MDCK cells were transiently transfected with VSVG3-SP-GFP. The cells were kept at 40°C to accumulate the protein in the ER. They were then infected with EPEC at 40°C for 3 hours, before VSVG3-SP-GFP was released to the TGN at 20°C for 2 hours. Finally, the infected cells were mounted on a time-lapse fluorescence microscope, and VSVG3-SP-GFP was released at 37°C. Time-lapse imaging was initiated after 15 minutes. **A.** Example of an uninfected cell and a cell infected with EPEC at initiation of imaging. Montages of the boxed areas are shown in B and the full time-lapse is shown in S2 Movie. Scale bar: 10 μm. **B.** Example of an EPEC microcolony (DIC, top panels) and VSVG3-SP-GFP localization (bottom panels) from a time-lapse sequence. Green arrows indicate the top of the EPEC colony, and black arrows indicate examples of EPEC bacteria. Scale bar: 3 μm. All images of GFP are shown as inverted contrast. **C.** Regions of interest were placed at EPEC colonies or at equivalent positions in non-infected cells. The fluorescence intensities of VSVG3-SP-GFP were quantified for the entire time-lapse sequences and normalized to t = 15 min. There was a statistically significantly higher level of recruitment VSVG3-SP-GFP to EPEC infection sites than equivalent positions on uninfected cells (*p<0.05 using repeated measurements analysis of variance). The measurements were performed on 25 infected and 25 non-infected cells. Standard deviation is indicated by the red and green regions.

Cells were infected with EPEC at 40°C for 3 hrs and the temperature was subsequently shifted to 20°C to accumulate a pulse of VSVG3-SP-GFP in the TGN. The release of the TGN VSVG3-SP-GFP pool was performed by mounting the infected cells on a time-lapse microscope at 37°C. Cells expressing VSVG3-SP-GFP were identified (Fig 5A) and imaged every minute for 80 minutes using time-lapse DIC and fluorescence microscopy (Fig 5B). To quantitatively assess the localization of VSVG3-SP-GFP upon release, the fluorescence intensities of GFP were quantified from regions of EPEC infection as wells as equivalent membrane regions on uninfected cells. As VSVG3-SP-GFP was released it appeared in the plasma membrane at sites of EPEC microcolony growth (p<0.05 using repeated measures analysis of variance) (Fig 5B and 5C and S2 Movie).

Since time-lapse imaging was initiated 15 minutes after starting the 37°C incubation, the initial phase of VSVG3-SP-GFP release was not acquired. To observe the initial phase of release, equivalent samples were fixed at 0, 10, 20, and 30 minutes after being transferred to 37°C. From these samples it was observed that slight accumulations of VSVG3-SP-GFP were present at the infection site 10 minutes after release. Following 20 minutes of temperature release of VSVG3-SP-GFP from the TGN, VSVG3-SP-GFP was observed at the EPEC microcolonies (S6 Fig).

Accumulation of VSVG3-SP-GFP at the infection site was observed as early as 10 min after release from the TGN, and time-lapse imaging showed that continuous VSVG3-SP-GFP recruitment was sustained for at least 95 min after release. This indicates that EPEC can recruit newly synthesized basolateral protein directly from the TGN, probably by redirecting basolateral vesicles from the TGN to the site of microcolony growth.

## Discussion

In the present study we showed that EPEC infection of MDCK cells altered cell polarity and that this occured locally at the EPEC microcolony rather than throughout the apical plasma membrane. Basolateral membrane proteins were re-directed and recruited to the site of microcolony growth at the apical membrane. The recruitment seemed to occur *via* redirection of the basolateral sorting machinery and thus, transport vesicles destined for the basolateral membrane.

EPEC colonies have been found to grow on top of cell-cell junctions, indicating that EPEC may have a preference for the basolateral membrane [62]. We investigated the infection efficiency of EPEC on MDCK cells displaying varying degrees of polarization and observed a clear preference for infection at sites where the basolateral membrane was exposed such as along the edges of wounded or subconfluent monolayers of MDCK cells. Furthermore, we saw that microcolonies were localized on top of cell juctions where three or more cells meet (tricellular tight junctions (tTJs) and multicellular junctions, respectively) in both semi-polarized and fully polarized monolayers. No colonies were observed on the free surface.

Supporting our findings, Aroeti *et al* also observed localization of EPEC on top of cellular junctions in fully polarized MDCK cells [62], although they did not report a preference for tTJs over cell-cell junctions. *Listeria monocytogenes* has been reported to attach and invade monolayers at multicellular junctions where extrusion of dead cells had exposed the lateral membrane [63].

Actin regultors such as WIP, alpha-actinin, Arp2/3, N-WASp, and Tks5 are normally involved in the formation of focal adhesions and/or podosomes in the basal cell region [64, 65]. However, during EPEC infection, these basolaterally localized actin regulators are recruited to the infection site [31, 66, 67]. We observed that transmembrane basolateral plasma membrane proteins not known to be involved in EPEC infection, AQP3-EGFP and the Transferrin receptor tagged with mCherry, were recruited to the infection site, where they accumulated at the EPEC microcolony. An apical protein, gp135, also reorganized at the EPEC microcolony with less intense labeling in regions of AQP3-EGFP recruitment.

The basolateral Exocyst vesicle tethering complex, Rab proteins and the basolateral v-SNARE, VAMP-3, localized at the site of microcolony growth. Interestingly, dominant-negative variants of two Rab proteins were not recruited. This suggests that basolateral proteins are specifically trafficked to the EPEC microcolony in the apical membrane by intracellular vesicle transport, possibly by rerouting of endosomes or transport of newly synthesized proteins directly from the TGN. This was supported by our findings that approximately 10–20 min after release from the TGN, the basolateral protein, VSVG3-SP-GFP accumulated at the microcolony.

Generally, basolateral proteins were still localized at the basolateral membrane upon infection and only a subset of proteins were recruited to the EPEC microcolonies. This indicates that the redirection of basolateral proteins was specific and not a side-effect of major cellular polarity disruptions.

In addition to recruitment of basolateral transmembrane proteins, EPEC has also been shown to affect plasma membrane lipids by inducing formation of phosphatidylinositol (3,4,5)-trisphosphate (PI(3,4,5)P_3_) at the infection site [68]. In polarized MDCK cells PI(3,4,5)P_3_ is restricted to the basolateral membrane domain where it functions as a regulator of basolateral membrane formation [69]. Collectively, the data presented in these studies and our results suggest that EPEC employs several strategies to recruit basolateral membrane proteins to the apical surface beneath the site of attachment.

In conclusion, we found that protein composition of the host cell plasma membrane surrounding individual EPEC and the microcolony is altered. EPEC had increased attachment when basolateral proteins were exposed at the plasma membrane. Rab proteins, the basolateral Exocyst complex, the basolateral v-SNARE VAMP3 were recruited, and basolateral VSVG3-SP-GFP rapidly accumulated at the EPEC microcolonies following release from the TGN. This indicates that basolateral transport vesicles are re-routed to the site of microcolony growth. Further investigation will clarify if recruitment of vesicles and accumulation of basolateral proteins aid EPEC microcolony growth by providing nutrients or perhaps facilitate better attachment sites.

## Materials & methods

### Bacterial strains, plasmids & antibodies

EPEC E2348 strain was kindly provided by Professor Gad Frankel, Imperial College London, UK. Rab5-GFP, Rab7-GFP, and Rab9-GFP, Rab5-DN-mCherry and Rab7-DN-mCherry were from Addgene. VSVG3-SP-GFP was kindly provided by Associate Professor Derek Toomre, Yale University, USA. The plasmid encoding Exo70-GFP was kindly provided by Team Leader Philippe Chavrier, Institut Curie, France. The plasmid encoding TfR-mCherry was kindly provided by Assistant Professor Matthew Kennedy, UC Denver, Colorado, USA. EPEC bacteria expressing GFP were produced by transformation of a GFP plasmid (GFP inserted into the pBluescript SK+ vector, kindly provided by Dr. Stefanie Bechstein, iNANO, AU, DK) into the EPEC E2348 strain and selected with 100 mg/L ampicillin. Before transformation, EPEC bacteria were made competent through a series of resuspensions in 10% glycerol using decreasing volumes after every spin.

Anti-gp135 mAb, clone 3F2/DB [70], kindly provided by Professor W. James Nelson, Stanford University, California, USA, was used as undiluted hybridoma supernatant. Antibodies against Lipid A were purchased from Abcam.

### Cell cultures

Madin-Darby canine kidney (MDCK GII) cells [71, 72] as well as MDCK cells stably expressing VAMP3-EGFP were obtained from Professor W. James Nelson, Stanford University, California, USA. AQP3-EGFP-MDCK cells have previously been published [73]. MDCK cells were cultured in DMEM with 1 g/L glucose (Gibco) supplemented with 10% (v/v) fetal bovine serum (FBS, Gibco) and a cocktail of 0.5 U/mL penicillin (Sigma), 0.5 g/mL streptomycin (Gibco), and 1 mg/mL kanamycin (Gibco). Cells were maintained at 37°C in a humidified atmosphere with 5% CO_2_.

MDCK cells were seeded using different techniques to create different levels of polarization. Cells were seeded on collagen-coated glass coverslips at subconfluency to generate cells with accessibility to basolateral proteins. To create semi-polarized monolayers, MDCK cells were seeded to confluency on collagen-coated coverslips and allowed to polarize for three days. To then yield access to basolateral proteins, the cell layer was wounded using a pipette tip (Fig 1E). To fully polarize MDCK cells they were seeded on collagen-coated 0.4-μm-pore Transwell filters and cultured for three to four days before infections with EPEC. Cells on filters were cultured in DMEM without serum in the apical chamber and DMEM with serum in the basal chamber in order to aid the polarization. Monolayers were given fresh medium in the basal chamber daily. Transepithelial resistance of the monolayers were measured with an STX3 electrode linked to an epithelial Voltohmmeter EVOM^2^ (World Precision Instruments). Finally, to create cell layers with intermixed apical and basolateral proteins, cells were seeded as instant confluent monolayers. Here, a surplus of cells were seeded in calcium free medium for one hour. The lack of calcium inhibits E-cadherin mediated transinteraction so the cells will be tightly packed without cell-cell junctions. Then, 1.8 mM CaCl_2_ was added to allow adherence junctions formation. After two hours, the cells had formed a monolayer without separation of apical and basolateral components [74]. Apical/basal cell surface polarity was tested for all conditions by monitoring AQP3-EGFP and GP135 by microscopy (S1 Fig).

For transient transfections, 100,000 MDCK cells were seeded on collagen-coated Transwell filter inserts in a 12-well plate or at 50% confluency on collagen coated coverslips. The cells were transfected in suspension with cDNA constructs using Lipofectamine 2000 (Invitrogen) according to the manufacturer’s protocol. Transiently transfected cells were cultured for two days prior to infection with EPEC to avoid declining expression of the Rab-GTPases and were therefore not as polarized as the three-day monolayers.

### EPEC infection of MDCK cells at different polarization levels

Before infection, cells were washed three times in plain DMEM to remove dead cells and traces of antibiotics. Cells were then infected with approximately 10^8^ EPEC bacteria (based on OD_600_) from a fresh overnight LB culture. The infection was performed in DMEM with 5% FBS and 5% LB medium for 5 min to allow adhesion of the EPEC bacteria. Cells grown on glass were then washed three times in plain DMEM to remove non-adherent bacteria and incubated at 37°C for four hours in DMEM with 5% FBS and 5% LB medium. Polarized monolayers were washed three times in 1xHANKS buffer and incubated at 37°C for six hours with 1xHANKS in the apical chamber and DMEM with 5% FBS and 5% LB in the basolateral chamber [35, 75–77]. Infected cells were fixed in 4% paraformaldehyde (PFA) for 10 min before immunofluorescent staining.

### Immunofluorescent staining and image analysis

After fixation in 4% PFA the cells were permeabilized for 5–10 min in 1xPBS with 0.1% Triton X-100. For labeling of EPEC bacteria, infected cells were stained with either hoechst, phalloidin-rhodamine, or anti-Lipid A antibodies. Cells were stained with 2 μg/mL hoechst diluted in 1xPBS with 3% BSA for 15–30 minutes to label EPEC and cell nuclei. EPEC actin pedestals were stained with 0.4 μg/mL phalloidin-rhodamine diluted in 1xPBS with 3% BSA for one hour at RT. Anti-Lipid A antibodies were used to label EPEC. Antibodies were diluted 1:100 in 1xPBS with 3% bovine serum albumin (BSA) and cells were stained for one hour at RT followed by three washes in 1xPBS and incubation with fluorescently conjugated secondary antibodies for one hour at RT. For staining of gp135, cells were incubated for 1 hour at RT with monoclonal antibodies against gp135 used as undiluted hybridoma supernatant. After incubation with secondary fluorescent antibodies, cells were washed three times and mounted on microscope slides using Glycergel Mounting medium (Dako). Images were captured using a Nikon T*i* Eclipse inverted fluorescence microscope, a Zeiss 200M inverted fluorescence microscope or a Zeiss LSM 700 confocal microscope. 63x or 100x oil-immersion objectives were used for image acquisition. Images were analyzed using ImageJ software [78] (available from NIH) and figures were assembled using Adobe Illustrator. Only Brightness/contrast were adjusted.

### Time-lapse imaging

For time-lapse imaging of initial EPEC attachment, MDCK cells seeded as semi-polarized monolayers on collagen-coated coverslips were mounted in a heating chamber and kept in phenolred-free DMEM medium with 10% FBS and 25 mM HEPES. EPEC bacteria were grown 2 hours in phenolred-free DMEM medium with 10% FBS and 25mM HEPES and subsequently, 5⋅10^6^ EPEC bacteria were added into the center of the heating chamber. Imaging was initiated directly after EPEC addition, and time-lapse sequences were taken every minute with DIC and the GFP channel. To observe recruitment of AQP3-EGFP, MDCK cells stably expressing AQP3-EGFP (AQP3-EGFP-MDCK) were seeded and infected in the same way. When one EPEC bacterium had successfully attached to the cell as observed by stationary behaviour for a few minutes, imaging was initiated. Time-lapse was performed with imaging every two min using three different z positions with DIC and GFP channels. Imaging was performed on a Nikon T*i* Eclipse inverted microscope equipped with an OkoLab heating chamber, Perfect Focus 3 system, a 100x objective, and an Andor Zyla cMOS camera.

### VSV-G pulse assay

MDCK cells were seeded subconfluently on collagen-coated coverslips and transfected with VSVG3-SP-GFP. The cells were incubated 16 hours at 40°C to allow the protein to be produced and accumulate in the ER. The cells were then infected as described above and incubated for another 3 hours at 40°C. Non-infected controls were incubated 19 hours at 40°C. The cells with or without EPEC infection were then incubated at 20°C for 2 hours to allow VSVG3-SP-GFP to be released from ER and retained in the TGN. Finally, VSVG3-SP-GFP was released at 37°C. For fixed samples, cells were infected for 4 hrs and after the temperature blocks moved to a 37°C incubator, fixed after 0, 10, 20, or 30 minutes and stained with hoechst as described above. Images were acquired on a Nikon T*i* Eclipse microscope using a 100x objective and an Andor Zyla cMOS camera.

For time-lapse imaging, the coverslips were mounted in an imaging chamber with 20°C phenolred-free DMEM medium with 10% FBS and 25 mM HEPES and placed in a 37°C imaging chamber in the microscope. Cells expressing VSVG3-SP-GFP were identified, and the medium changed to 37°C medium and time-lapse imaging initiated, as described above. A total of 25 infected cells and 25 uninfected cells were imaged on three days. Intensity of VSVG3-SP-GFP was quantified using ImageJ [78]. Intensity over time was measured in a region of interest around an EPEC colony or an equivalent plasma membrane region on an uninfected cell. Because of varying expression levels in transiently transfected cells, the fluorescence intensities were normalized to the first measurement.

### Statistics

All experiments were performed at least three times. The numbers of EPEC infecting semi-polarized and instant confluent monolayers were compared using Student’s t-test. The quantifications of VSVG3-SP-GFP were statistically compared using repeated meaures analysis of variance (ANOVA). Statistics analyses were performed in MS Excel and MATLAB (MATLAB Release 2015b, The MathWorks, Inc., Natick, Massachusetts, United State).

## Supporting information

S1 Fig Distribution of apical and basolateral proteins in subconfluent and semipolarized MDCK cells. AQP3-EGFP-MDCK cells were seeded on collagen coated glass coverslips to obtain subconfluent cells or instant confluent monolayers. At specific growth spans, cells were fixed and stained with anti-gp135 antibodies. Subconfluent cells (top left panel) were fixed after 1 day of growth while instant confluent monolayers were fixed at time points ranging from 2 hrs (instant confluent monolayer, wounded and instant confluent monolayer) to 3 days (instant confluent monolayers + 1, 2, and 3 days). Images of basal, middle and apical sections were taken; shown are inverted contrast of hoechst labelled nuclei (left column), AQP3-EGFP (second column from the left) and gp135 immunostaining (third column from the left). In the overlaid images (right column) AQP3-EGFP signal is depicted in green, gp135 staining is shown in red and hoechst in blue. Scale bar: 10 μm.
(EPS)

S2 Fig EPEC infection pattern on unpolarized versus fully polarized cells. MDCK cells were seeded on filter supports as instant confluent monolayers and allowed to polarize for 4 days (bottom panels) or not allowed to polarize (top panels). The cells were infected with EPEC for 4 hours and fixed. Images are inverted contrast of hoechst labeling nuclei and EPEC bacteria. Scale bar: 10 μm.
(EPS)

S3 Fig The transferrin receptor is recruited to EPEC microcolonies. Subconfluent MDCK cells were transiently transfected with mCherry linked transferrin receptor (TfR-mCherry). The cells were infected with EPEC for 4 hours, fixed and stained with hoechst to label cell nuclei and EPEC bacteria. Arrows indicate examples of EPEC with TfR-mCherry recruitment. Scale bars: 10 μm and 3 μm for inserts.
(EPS)

S4 Fig Rab proteins localized in a punctuate, heterogeneous pattern in the cytoplasm. MDCK cells were transiently transfected with Rab5-GFP, Rab5-DN-mCherry, Rab7-GFP, and Rab7-DN-mCherry. The cells were fixed and stained with hoechst to label nuclei. Hoechst is red in merges, whereas the Rab proteins are shown as green. Scale bar: 10 μm.
(EPS)

S5 Fig Localization of vesicle docking machinery components. **A.** MDCK cells transiently transfected with Exo70-GFP were fixed and stained with hoechst to label nuclei (red in merge). **B.** MDCK-VAMP3-EGFP cells were polarized on semi-permeable Transwell filters, fixed and stained with hoechst to label cell nuclei. Positions of xy sections and xz projections are indicated by white lines. Scale bars: 10 μm.
(EPS)

S6 Fig VSVG3-SP-GFP localization upon release from a TGN temperature block in fixed samples. MDCK cells were transiently transfected with VSVG3-SP-GFP. The cells were kept at 40°C to accumulate the protein in the ER. They were then infected (A) or left uninfected (B) with EPEC at 40°C for 3 hours, before VSVG3-SP-GFP was released to the TGN at 20°C for 2 hours. Finally, VSVG3-SP-GFP was released at 37°C for 0, 10, 20, or 30 minutes as indicated. Cells and bacteria were then fixed and stained with hoechst (upper panels, shown as inverted contrast). VSVG3-SP-GFP is shown in the second panels as inverted contrast; VSVG3-SP-GFP still retained in TGN was observed at all timepoints (yellow arrowheads), and clear recruitment to the infection site was observed after 20 minutes. Arrows indicate examples of EPEC bacteria. Scale bars: 10 μm and 3 μm for the insets.
(EPS)

S1 Movie Timelapse DIC imaging of attachment of an EPEC bacterium to the surface of a cell. EPEC movement to a tricellular cell junction, and establishment of a microcolony.
(AVI)

S2 Movie Timelapse DIC and fluorescence imaging of VSVG3-SP-GFP release in a cell with or without EPEC infection. Montages from the same image sequences are shown in Fig 5. Time stamps in minutes are indicated in the top left corner.
(AVI)
